# Electrical Heaters for Anti/De-Icing of Polymer Structures

**DOI:** 10.3390/polym15061573

**Published:** 2023-03-22

**Authors:** Aleksei V. Shiverskii, Mohammad Owais, Biltu Mahato, Sergey G. Abaimov

**Affiliations:** Center for Petroleum Science and Engineering, Skolkovo Institute of Science and Technology, Bolshoy Boulevard 30, bld. 1, Moscow 121205, Russias.abaimov@skoltech.ru (S.G.A.)

**Keywords:** anti/de-icing, composites, nanoparticles, Joule heating, thermal conductivity, electro conductivity, percolation threshold

## Abstract

The problem of icing for surfaces of engineering structures requires attention more and more every year. Active industrialization in permafrost zones is currently underway; marine transport in Arctic areas targets new goals; the requirements for aerodynamically critical surfaces of wind generators and aerospace products, serving at low temperatures, are increasing; and fiber-reinforced polymer composites find wide applicability in these structural applications demanding the problem of anti/de-icing to be addressed. The traditional manufacturing approaches are superimposed with the new technologies, such as 3D printers and robotics for laying heat wires or cheap and high-performance Thermal Sprayed methods for metallic cover manufacturing. Another next step in developing heaters for polymer structures is nano and micro additives to create electrically conductive heating networks within. In our study, we review and comparatively analyze the modern technologies of structure heating, based on resistive heating composites.

## 1. Introduction

Fiber-reinforced polymer composites (FRPC) have found wide demand in structural applications. Now, they are an integral part of many engineering solutions. In comparison with metals, polymer composites have superior mechanical performance, and reduced weight; they are less susceptible to fatigue and more corrosion resistant. The use of composites allows for manufacturing products with complex shapes, which reduces the number of parts, increases cost-effectiveness and reliability, and speeds up the assembly of products. As only one example, the implementation of FRPC technologies for blades of wind turbines has revolutionized the wind power industry, increasing the power output from the range of 2–3 MW to more than 12 MW due to the increase in blades’ size [1]. Another example is the high-end applications of the carbon/epoxy FRPC, the so-called “black aluminum”, in the aerospace industry [2]. Currently, the share of composite elements in the design of a modern aircraft reaches up to 50% [3,4,5]. The third example comes from marine vehicles where polymers find wide applicability [6]. The unique properties including thermal conductivity [7], electrical conductivity [8], and transparency to various types of radiation [9], are also highly demanded in various design applications.

Nowadays, new approaches in advanced structures and smart materials require material to conduct not only its primary mechanical or functional role, but to be multi-purpose, simultaneously addressing several demanded functionalities of the in-service support for a structure. One such task is to provide de/anti-icing of the working structural surfaces [10]. The formation of ice on hard surfaces can cause huge economic damage to society and poses a great danger [11]. The ice crust formation on the surface of an aircraft limits its performance [12], can significantly change the dynamic flight characteristics, and even lead to flight accidents [13,14]. Ice accumulation on ocean-going ships can change their balance and affect their safety [15]. The operation of wind turbines directly depends on the condition of the surface of the blades [16].

In general, ice formation can occur not only on FRPC, but on different surfaces, and cause a number of problems in everyday life, such as ice formation on roads and footpaths [17], on power line wires [18], and bridge cables [19], on systems of air recirculation [10] and roofs [20], etc. In all these cases, the formation of ice creates a negative impact on engineering structures or poses a danger to human life.

Thereby, it is important to develop effective heating methods that can protect working surfaces from ice formation. De/anti-icing functionality can be integrated into a composite part design. This problem is of special importance due to the necessity of operations in the Arctic and Antarctic regions.

A lot of anti/de-icing technologies are available on the modern market. Some of them can be widely used, whereas others are only for special applications. The most famous solutions are electrothermal [21], photothermal [22], ultrasonic [23], hydrophobic [24], and chemical [25]. Our research contains the review and comparative analysis of the modern de/anti-icing technological solutions targeting FRPC-based electrical heaters.

**Figure 1:**
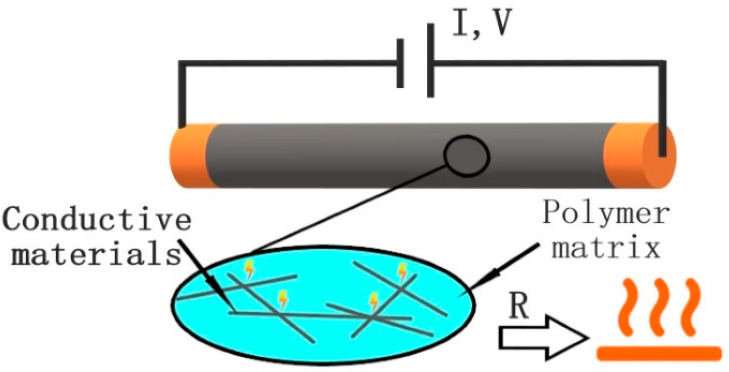
Schematic of Joule heating process.

These electrical heaters work on the principle of Joule heating. It is also known as Ohmic or resistive heating as the heat generated during Joule heating depends on the resistance and is calculated by Ohm’s law of P = I^2^ R, where P is power, I is current, and R is resistance [26]. The phenomenon of Joule heating occurs when charge carriers, typically electrons, interact with the conductive materials body. An electric field is created by a voltage difference between two points in the conductor, which accelerates the charge carriers in the direction of the electric field and gives them kinetic energy, Figure 1. As these charged particles collide with the quasi-particles in the conductor, which are the ionic lattice oscillations in a crystal’s harmonic approximation, energy is transferred from the electrons to the lattice, creating further lattice oscillations. The radiation or thermal energy measured in an experiment originates from these ion oscillations. This process serves as the foundation for numerous practical uses of electric heating.

## 2. Foreign-Object Heaters for FRPC

### 2.1. Metal Foils and Grids as FRPC Surface Heaters

The first steps in the implementation of the resistive heating of an FRPC part surface to prevent ice formation were carried out by placing an electrothermal material (heater) on the surface, with the heater being a type of functional electrical resistor that can convert electrical energy into heat.

In one of the first industrial studies on anti/de-icing [27], several solutions were tested as a heater: etched metal foil grid, sprayed metal grid, knitted metal wire/glass fabric, a pierced expanded metal grid, and wires integrated into rubber. Different types of heaters were placed between the erosion shield material (Nickel alloy) and FRPC blade structure. Although this study demonstrated great future promise for the use of active ice removal systems, it also identified the common polymer burnout problem arising from excessive wire temperatures. Besides, the developed heaters, as external to the FRPC part, were likely to short-circuit against the conductive protective erosion shield material. From the current perspective, electrothermal anti/de-icing systems, which were used in the 70s in the form of thermal spacers, electrically heated foil, or electric heating elements made of metal or carbon fiber on the surface of FRPC products, were bulky, expensive, and often degraded the aerodynamic performance of the product.

The placement of a heater on a surface of an FRPC structure is possible either on the internal side or on the external side. In [21], it was demonstrated that implementing an electrical heating element on the internal side of a composite structure led to increased power consumption due to the high temperature differences between the heat application surface and de-icing surface, separated by the FRPC laminate, which made this approach unprofitable. The metal foil heaters showed the best results when they were placed on the external surface of an FRPC structure [28].

### 2.2. Metal Coatings as FRPC Surface Heaters

The early external heaters of FRPC structures were difficult to use and not effective. Later, engineers found ways to increase their capabilities. Nowadays, one of the top ways to create a cheap and repairable heating coating on an FRPC surface is by thermal spraying technology [29].

In [30], the authors applied a flame-sprayed nickel-chromium (NiCr) coating on the FRPC surface for use as a heating element. Application of the coating with high-temperature thermal technology was shown not to destroy the integrity and mechanical properties of the FRPC laminate due to the implementation of a protective sand-epoxy layer. The resulting coating was found to provide uniform heating. Testing showed that when cooled to −25 °C, the FRPC surface temperature maintained above 0 °C. The technology of thermal spraying of metal films also allows for the application of coatings to FRPC surfaces of complex geometries and the repair of damaged coatings [31]. The current system used on Boeing 787, requires a steady state temperature of 6 °C for effective anti-icing under −18 °C operational ambient conditions, expending 11.8 kW/m^2^, not taking into account the energy absorbed by the composite structure itself [32]. Moreover, the deposition of metal layers on the polymer can be conducted by other technologies including physical vapor deposition [33], chemical vapor deposition [34], and plasma-enhanced chemical vapor deposition [35]. These methods are relatively expensive and not suitable for manufacturing thick metal coatings (over 100 μm) at high deposition rates [36]. Nevertheless, they allow for obtaining layers from non-traditional materials, such as transparent and electroconductive indium tin oxide or extra-thin metal films. However, for transparent applications, it is more interesting to use systems based on thin layers of single-wall carbon nanotubes (CNT) [37].

### 2.3. Metal-Based Heaters Imbedded into FRPC

**Figure 2:**
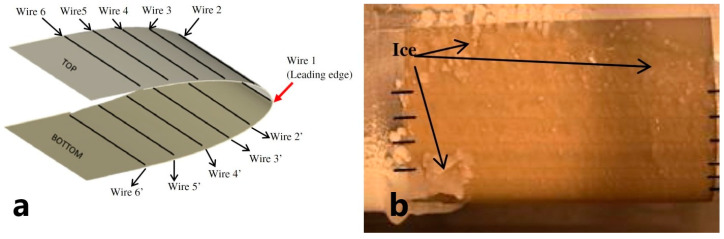
(**a**) Schematic of a composite with integrated thermal elements, (**b**) De-icing behavior of the composite in icing test. (Reprinted with permission from Elsevier, copyright 2013) [40].

A large area of research in anti/de-icing was devoted to the placement of heating elements embedded into an FRPC product. However, heater implantation into an FRPC product may lead to the degradation of its functional or mechanical properties, especially interlaminar. In [38], the authors demonstrated that the implantation of a foil as a heater led to the development of delamination in the FRPC part under high loads. However, in [39] this drawback was not observed for perforated metal foils as contact pads supplying electrical current to other types of heating elements inside the FRPC. Authors in [40] presented a numerical and experimental development of the concept of a thermoelement based on NiCr wires to be embedded into FRPC profiles of wind turbine blades as an active anti-icing system, Figure 2a. It was experimentally shown that the edge region of the profile was the most susceptible to icing due to the maximal convective heat transfer over this region and the fluid load, Figure 2b. For anti-icing in cold and dry conditions, the temperature at the leading edge was kept at 60 ± 3 °C for low wind speed. The minimum surface temperature of the rest of the FRPC profile was maintained at 26 °C. The power consumption of the system was 8.3 kW/m^2^, which is lower than 9.2 kW/m^2^—the power consumption for a similar aluminum profile with outside heaters put to the same icing conditions.

Another interesting case is to combine embedded heating elements with electrically conductive fibers in a fiber metal laminate (FML) as an FRPC structure [41]. The FML systems are widely used, and their production technologies are well studied. The use of such a combined anti-icing system can lead to a decrease in product weight, especially for outdoor structures. In [42], heated glass laminate aluminum-reinforced epoxy composite structure (GLARE) was studied as one of the most widely utilized FMLs. Since in [38] it was shown that metal foil embedded into an FRPC can cause delamination, for the GLARE as a serial product it was necessary to demonstrate that an embedded heater does not worsen mechanical properties; in particular, the absence of linear viscoelastic creep. Authors of [42] showed that the metal layers and glass fibers in GLARE offset the effect of interlaminar creep in the heated state. Continuous physical aging slows down this process in long-term temperature and stress loading. The overall creep effect is thereby limited, which leads to applications of heated GLARE in FRPC structures [43,44].

**Figure 3:**
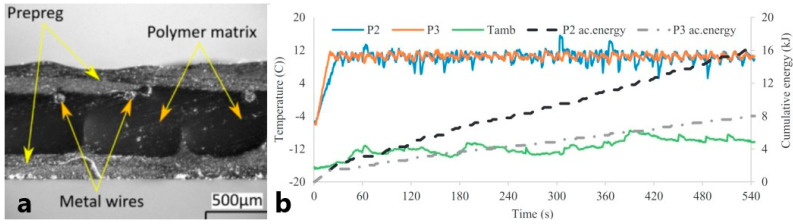
(**a**) Cross-section microscopic image of the 3D printed heater, (**b**) average temperature of two panels and ambient temperature with the cumulative energy of both panels (Reprinted with permission from Elsevier, copyright 2019) [45].

In studies [40,41,42,43,44] discussed above, the process of embedding the heating elements into the FRPC structures was time- and effort-consuming. It can suffer from manufacturing inconsistencies and human errors. [45] proposed to introduce 3D printing to automate the manufacturing process. The authors used continuous NiCr wire and thermoplastic as 3D printing material to create a heater embedded into an FRPC plate. The NiCr-heaters in thermoplastic volume were printed by a meander pattern without a gap (i.e., as a continuous filament). A meander pattern was chosen as providing an evenly distributed heat flux on the surface of the FRPC plate. Then, heating plates were covered with a layer of Kapton film for electrical insulation. The assembled heater was placed between two sheets of carbon fiber prepregs and cured in a vacuum bag in an oven at 100 °C, Figure 3a. Obtained FRPC plates were field-tested onboard a marine vessel at subzero temperatures in the sea. The heater effectively kept the surface of the FRPC panel free of ice. Temperature distribution across the FRPC panel was uniform and stable in time: P2 and P3 curves in Figure 3b. Experimental studies at an ambient temperature of −20 °C showed anti-icing protection at the power consumption of this system of 10 kW/m^2^.

The application of metal-based heaters integrated into FRPC looks promising. Moreover, optimization of the manufacturing process, thanks to the possibility of 3D printing to lay the wire inside the product, allows for reducing the risks of malfunction. Nevertheless, embedded metal heaters have a big disadvantage—they are nearly impossible to be repaired.

### 2.4. Carbon-Based Heaters Imbedded into FRPC

**Figure 4:**
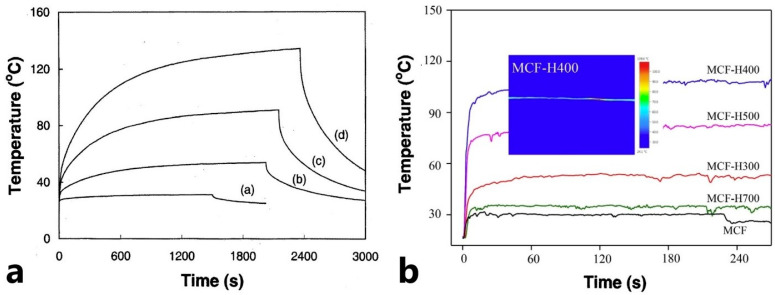
(**a**) Changes of T_max_ for pure carbon fibers mat at (a) 4 V, (b) 8 V, (c) 12 V and (d) 17 V (Reprinted with permission from Elsevier, copyright 2003) [51], (**b**) changes of T_max_ of the MCF as a function of heat treatment temperature, at 12 V (Reprinted with permission from Elsevier, copyright 2019) [52].

As an alternative to embedding foreign materials as heating elements into FRPC, the possibility is often present to imbed FRPC-related materials, for example, FRPC reinforcing elements, as heat sources. Historically, this approach was developed in parallel with external heaters. One of the pioneering works in this area confirmed the possibility of using carbon fibers as heaters. In [39], the authors manufactured an FRPC heater based on industrial carbon fibers and resin; in this case, the mesh from nickel was used as electrical contact pads. However, the developed heater possessed low efficiency and high heterogeneity of the generated heat field on the surface of the FRPC. On a 10 cm segment, the temperature drop was 15 °C. Moreover, the authors had to overcome the difficulty of making electrical contacts with carbon fibers. Currently, these problems are solved by functionalizing the surface of carbon fibers using the electroconductive sizing of Ni, Cu, Zn, Pt, Ag, or their alloys [46,47,48,49,50]. A modern study of carbon fibers showed their excellent properties as heaters [51]. On samples from carbon fiber mats, heating up to 140 °C has been achieved, at an applied voltage of 6.5 kW/m^2^, d-curve in Figure 4a.

In [52], the researchers investigated the dependence of the electrical conductivity on the post-annealing temperature of metalized carbon fiber (MCF). The effect of the annealing temperature of MCF on the heating properties of FRPC is shown in Figure 4b. The existence of temperature dependence makes it possible to expand the field of application of carbon fibers as heaters and allows more precise tuning of the characteristics of the FRPC composite. Comparing the graphs in Figure 4, we can suggest that the heating systems based on pure carbon fibers are better for scenarios where the high heating temperature is more important than the heating rate, and the MCF system is better for scenarios with a high heating rate.

**Figure 5:**
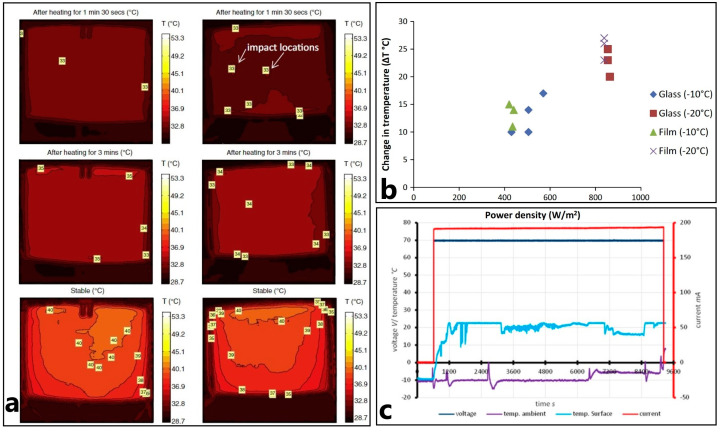
(**a**) Thermal imaging before and after impact on ECT panel, (**b**) change in temperature as a function of power density for anti-icing studies (reprinted with permission from Elsevier, copyright 2015) [55], and (**c**) de-icing test graph at −5 °C and wind speed of 7 m/s (Reprinted with permission from John Wiley and Sons, copyright 2017) [57].

Some studies considered the possibility of using electrically conductive carbon textiles as a heating layer in FRPC [53,54,55,56]. In [55], a commercially available electro-conductive carbon-based textile (ECT) by “Gorix” was used for FRPC heater manufacturing. The aim of the work was to investigate how the low-velocity impact on the FRPC heater influences the anti/de-icing properties. The FRPC heater based on ECT material was damaged at two locations, Figure 5a, but it continued to function and demonstrated a good heating performance after impact, comparable to 90 % of heat flow from the non-damaged counterpart. In the anti-icing test at a chamber temperature of −20 °C, it showed low energy consumption, only 0.9 kW/m^2^ (Figure 5b), and the ice melting time at the same power in the de-icing test was 40 min.

Besides fibrous carbon materials, electrically conductive carbonaceous nanofillers in the form of a mat were also utilized for FRPC heaters. In [57], the authors proposed using a multi-walled carbon nanotube (MWCNT) mat as an active heat element of an FRPC heater. For mat manufacturing, MWCNTs were typed into a non-woven textile substrate by rotary screen-printing. CNTs in the mat did not possess an orderly structure and presented isotropic and homogeneous 3D mesh. MWCNT mat was embedded into an FRPC wind turbine blade manufactured by vacuum infusion technology. The FRPC wind turbine blade was tested for de-icing in the climatic chamber at −5 °C with a simulated wind speed of 7 m/s. For this case, the FRPC heating elements melted in 25 min a layer of ice with an energy consumption of 1.33 kW/m^2^; the process dynamics are shown in Figure 5c.

**Figure 6:**
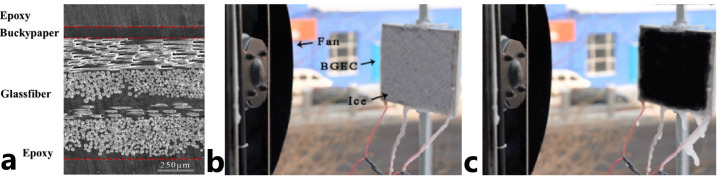
(**a**) SEM image of the cross-section of the buckypaper-FRPC heater, and (**b**) image of buckypaper-FRPC heater before (**c**) and after de-icing at −22 °C and wind speed 14 m/s (Reprinted with permission from Elsevier, copyright 2014) [60].

Thin carbon nanotube films with entanglement keeping CNTs in place are commercially available and are called carbon nanotube paper or buckypaper. Their utilization allows for solving the problem of CNTs dispersion during vacuum infusion [58]. Thanks to the use of buckypaper, CNTs as integrated heaters have become even more widespread today [58,59,60] as no additional complex operations, such as on-ply printing, are involved in manufacturing. Moreover, the absence of additional operations at CNTs allows for preserving their high physical and mechanical characteristics [61]. Additionally, the structure of the buckypaper is porous, and this is very convenient for impregnation with a polymer. The possibility to utilize buckypaper as a heated element of an FRPC heater was studied in [60], Figure 6a. The de-icing test showed the possibility of effectively using the FRPC structure at −22 °C and strong wind, up to 14 m/s. The ice melting time was 7 min at a power consumption of 11 kW/m^2^, Figure 6b,c.

**Figure 7:**
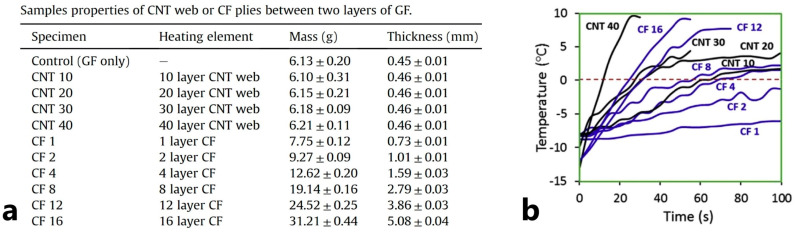
(**a**) Properties of samples with CNT and CF plies, (**b**) de-icing test graph at 16 V (Reprinted with permission from Elsevier, copyright 2018) [62].

The efficiency of an FRPC heater based on buckypaper was compared with the efficiency of an FRPC heater based on carbon fibers at identical conditions [62]. The buckypaper was produced by pulling CNTs from a nano-forest (aligned array of MWCNTs), synthesized by the chemical vapor deposition process. The authors claimed that using buckypaper layers as heating sources made it possible to obtain up to four times lighter FRPC heaters than their carbon fiber analogs, Figure 7a. The experimental study showed that the heaters based on 40 buckypaper layers were capable of effective de-icing. In the test at an environmental temperature of −12 °C, the de-icing process took 15 s. with an FRPC heater power up to 4.9 kW/m^2^ vs. 25 s and 6.5 kW/m^2^ for 16 carbon fibers layers, Figure 7b.

**Figure 8:**
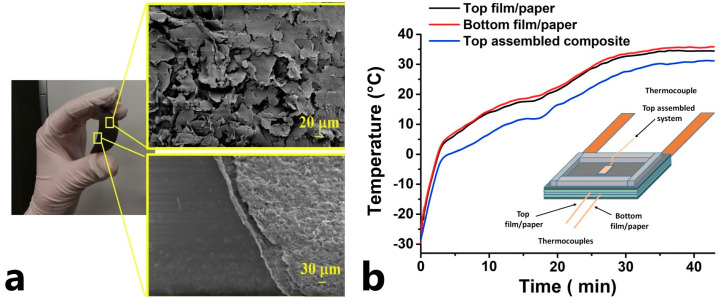
(**a**) SEM images of the graphene paper, (**b**) the temperature profile during the de-icing process, at an environmental temperature of −32 °C (Reprinted from Elsevier, copyright 2019) [63].

As a competitor to buckypaper, carbonaceous sheets based on graphene (graphene paper) and graphite (graphite paper) were also studied, Figure 8a. In [63], the FRPC heater was assembled on the base of a graphene paper, Figure 8b. It performed the de-icing task successfully and heated the surface from −32 to 0 °C in 4 min at power up to 1.6 kW/m^2^. This power allowed the FRPC surface to heat up to 37 °C during the next 40 min.

**Figure 9:**
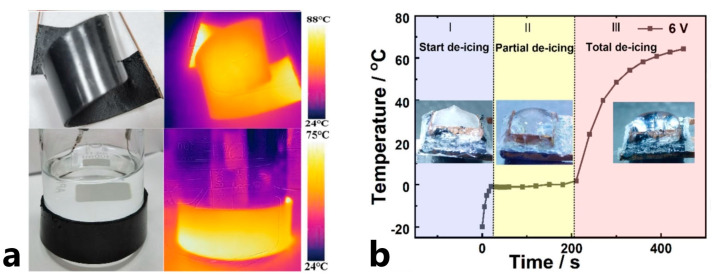
(**a**) Photography of flexible FRPC, (**b**) time-dependent temperature profiles of the FRPC at 6 V (Reprinted with permission from Springer Nature, copyright 2021) [64].

Authors in [64] developed a more complex design: they manufactured a flexible FRPC from several graphene nanosheet papers (CNSs) and styrene-butadiene rubber (SBR) layers, Figure 9a. The SBR polymer is flexible and possesses both high thermal conductivity and adhesive properties. The heating temperature of FRPC heaters based on CNSs@SBR reached 142.9 °C at a bias voltage of 6 V with low power of around 2.1 W and was stable after fatigue loading of 4000 folds. The de-icing tests proved that the flexible FRPC heater is efficient and competitive for de-icing applications when a high de-icing rate is required. At an ambient temperature of −20 °C, the ice melt time was 210 s, Figure 9b, and the power consumption was 3.6 kW/m^2^. The flexibility of the FRPC heater based on CNSs@SBR allows for its use on flexible as well as complex shape surfaces with huge areas for anti/de-icing tasks.

### 2.5. Comparative Analysis of the Foreign-Object Heater Technologies

**Figure 10:**
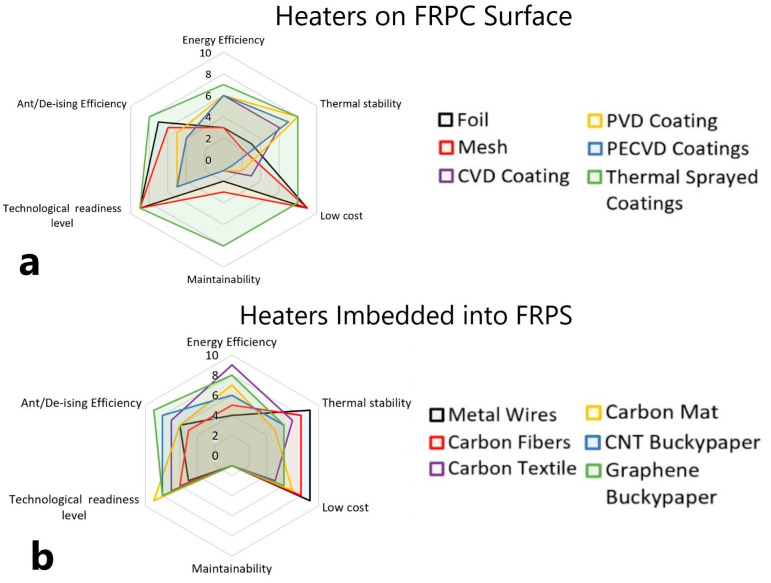
Comparison of the efficiency of heaters (**a**) placed on FRPC surface, and (**b**) embedded into FRPC, fabricated by different technologies.

The comparison of different heaters technologies is presented in Figure 10 and in Table 1. The analysis of six main criteria shows that in the case of heaters on FRPC surfaces, Figure 10a, the thermal sprayed technology allows for obtaining the best solution. For the case of embedded heaters, Figure 10b shows that carbon textiles, CNT, and graphene buckypapers allow for obtaining heaters with the best properties and low cost. The carbonaceous nanofiller mat heaters have several advantages: high thermal- and electro-conductivities, sustainability, simplicity of implementation, and improved mechanical properties of FRPC. However, heaters based on these materials have low maintainability: the impossibility of easy repairs due to embedment.

From this point of view, nowadays thermal sprayed technology is considered the most applicable option for mass production since these heaters for FRPC have high maintainability due to accessible surface coating, are easily manufactured, and allow for obtaining good thermal characteristics at low cost.

The foreign-object heaters have been around for a while and come in various types. However, existing solutions became widely applied in FRPC heaters production only recently. Early solutions had low efficiency, were difficult to manufacture, and had a high cost. Modern technologies, efficient and commercially viable, to produce metal alloy surface heaters for FRPC have been developed with the help of advanced manufacturing technologies based on robotic systems. Currently, the main disadvantages of metal alloy surface heaters are that they create an additional load on the FRPC and are prone to the risk of electric shock. We expect that the next step in the development of heaters for FRPC surfaces will be based on the implementation of self-heating polymer compositions. Compared to metal coatings, the technologies for the application of polymer coatings on the FRPC are cheaper and easier. In some cases, polymer coatings improve the mechanical properties of FRPC and give additional functionality. Self-heating coatings are discussed in more detail in Section 4.

The main disadvantage of embedded heaters for FRPC, unlike surface ones, has always been the complexity or even impossibility of their repair. This fact greatly hindered their development. However, a large amount of research and development of technologies to produce nanoscale materials has opened new opportunities in this approach. The main advantage of using heating elements manufactured from nanoscale materials is the extremely high reliability, since even with significant damage they continue to remain operational. Moreover, embedded nanoscale heaters are integrated into FRPC and do not add extra weight or load, instead, they enhance the material’s mechanical properties, unlike metal alloy surface heaters. However, new technologies obtaining heating elements from nanoscale materials with given characteristics have just started developing. Manufacturing heaters based on nanoscale materials for large-area RFPC is expensive and not scalable so far. This area of research is actively developing and has great prospects, especially in improving the physical properties of heating elements from nanomaterials.

An important parameter of FRPC is the thermal conductivity of the matrix since the efficiency of heat depends on it. The increase in the thermal conductivity of the matrix can be achieved by adding nanosized materials to its FRPC composition. The influence of various additives on the thermal conductivity polymer matrix is discussed in more detail in the next chapter.

## 3. Thermally Conductive Polymers for FRPC Matrix

Improvement of the heat conductivity of polymers by nanofillers can also play an important role in enhancing heater performance. The matrix in composite materials ensures the solidity of the material, and the transmission and distribution of stress in the reinforcement determine the heat, moisture, fire, and chemical resistances [68]. The rate of transfer of thermal energy from the internal heater to the outer surface of the composite product directly depends on the thermal conductivity of the matrix material.

Standard polymers used in the manufacture of FRPC products have low thermal conductivity *k*, usually in the range from 0.18 to 0.44 W/mK [69,70]. The addition of high-*k* nanoparticles (boron nitride, CNT, graphene, etc.) to polymers improves the thermal conductivity of the matrix by several times [71,72]. However, electrically conductive nanoparticles, such as CNTs or graphene, cannot be used in combination with embedded heaters as they transform the whole composite into an electrical conductor and lead to a short circuit inside the structure. Thereby, only dielectric nanofillers are considered in this section. The electrically conductive additives will be reviewed in the following section.

**Figure 11:**
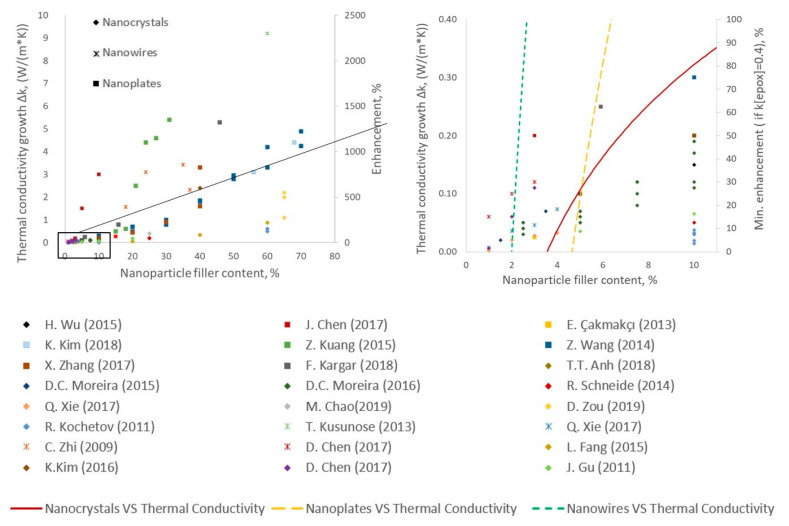
Thermal conductivity properties of dielectric nanoparticles [50,78,81,82,83,84,85,86,87,88,89,90,91,92,93,94,95,96,97,98,99,100], full-scale comparison and close-up. Three trends compare influence of 0D, 2D, and 1D particles.

The use of nanoparticles depends on the possibility of uniform dispersion and distribution of the filler on a submicron scale [73], which is an essential requirement for fiber-reinforced composites where typical fiber diameters and the gap in between them are in the range of microns. The limitations of nanoadditive utilization include agglomeration, bundling, anisotropic orientation distribution, entanglement, filtration on fibrous reinforcement [74], provoking stress concentrations, and degradation of target characteristics. The review of the results of experimental works on improving the thermal conductivity of a polymer by adding dielectric nanoparticles is presented in Figure 11. The nanofillers are divided into three groups: spherical particles (0D materials)—mainly oxides nanocrystals; materials with a high aspect ratio (1D materials)—structures such as nanowires; and nanoplates (2D materials).

Figure 11 shows that the smallest increase in *k* is in general achieved by nanocrystals (rhombus markers), for which the maximal investigated Δk of the nanocomposite rarely exceeds 1 W/mK. This agrees with the low ability to form a percolation network for a particulate composite [75]. Nanowires are expected to be more prone to percolate than nanoplates. Trend lines plotted for each group (Figure 11) show that nanoplates (squares markers) have the same behavior of dependence thermal conductivity from filler, as nanowires (asterisk markers). However, in the case of nanowires, the thermal conductivity starts to increase earlier. According to Figure 11, nanowires and nanoplates are the best fillers for thermally conductive nanocomposites since their Δk ranges largely overlap. However, liquid composite molding technologies for the production of polymer composites with fibrous reinforcement impose restrictions on the use of 1D and 2D nanomaterials due to the filtration effect that occurs during the impregnation of reinforcing elements [76,77]. In [78,79,80] the use of more than 10 wt.% additives was shown to lead to entrained air, aggregation of nanoparticles, and poor interfacial contact between the polymer and particles, limiting the additional improvement in thermal conductivity. Moreover, this high wt.% of additives leads to significant changes in other material properties or causes their degradation.

Taking into account the above factors, the use of nanoadditives for polymer matrix in FRPC is typically limited to 10 wt.% and excludes particles with high aspect ratios. As presented in the zoomed region in Figure 11, ceramic fillers such as alumina, aluminum nitride, silicon carbide, and boron nitride are most often used as fillers for making FRPC that possess both high electrically insulating properties and increased thermal conductivity. Further, we will discuss this class of materials in detail.

Alumina (Al_2_O_3_) is widely used in electrical engineering due to its excellent electrical insulation, ideal thermal conductivity of up to 25 W/mK, and low cost [101]. Authors in [93] showed that by adding 10 wt.% Al_2_O_3_ nanoparticles, the *k* value of the Al_2_O_3_/epoxy resin composite was increased from 0.2 to 0.39 W/mK (by 95%). Further, the addition of 70 vol.% of Al_2_O_3_ nanoparticles increased the value of k to 13.46 W/mK in [102].

**Figure 12:**
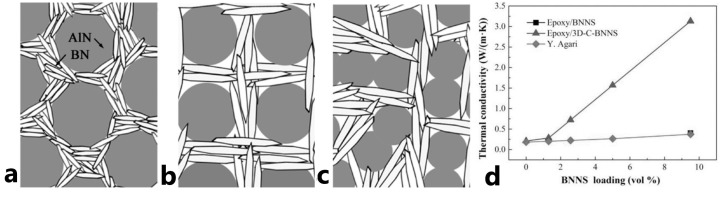
Schematic image of the BN@AlN systems in relation to (**a**) 2:1, (**b**) 1:1 (**c**) 1:2 (Reprinted with permission from Elsevier, copyright 2012) [104], and (**d**) thermal conductivity of composite based on BN nanosheet at room temperature (Reprinted with permission from John Wiley and Sons, copyright 2016) [87].

Aluminum nitride (AlN) is widely used as an electronic ceramic substrate and thermally conductive sealing material because of its high thermal conductivity of up to 230 W/mK, excellent heat resistance, good electrical insulation, and high permittivity for ceramics [103]. At low concentrations, up to 10 wt.%, AlN showed weaker result than Al_2_O_3_, increasing *k* by nearly 10% on 0.02 W/mK [99]. In [104], the authors considered a hybrid nano-filler approach with the possibility of using AlN nanoparticles as bricks for building a 3D network from boron nitride (BN) nanorods, Figure 12a–c. A 3D network of BN allows for increasing the thermal conductivity of epoxy resins, delivering a maximum value of 8.0 W/mK with a 1:1 ratio and 80 vol.% (resin only 20 vol.%), due to the orientation of BN nanoplates perpendicular to each other (Figure 12b).

Silicon carbide (SiC) has a low price, high thermal conductivity of up to 200 W/mK, and high wear resistance [100]. The *k* value of the SiC/epoxy composite was increased to 0.33 W/mK due to the addition of 3.0 wt.% SiC, thereby increasing the thermal conductivity of the SiC/epoxy composite by 50% [95]. SiC/polystyrene composites were prepared by hot pressing [105] while increasing *k* by 300% from 0.182 to 0.566 W/mK by adding 32.8 vol.% SiC.

**Figure 13:**
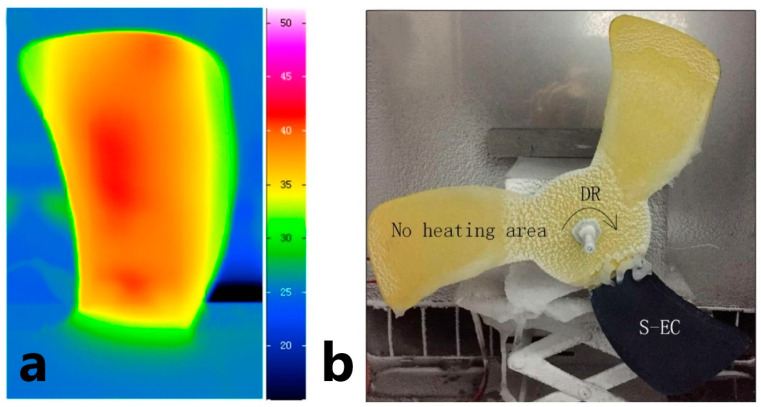
The application of the coating on rotating blade surface. (**a**) Infrared image of the blade coated with an electric heating coating (S-EC) supplied by direct current voltage. (**b**) The images after anti-icing test; DR represents the direction of rotation (Reprinted with permission from Elsevier, copyright 2018) [158].

Boron nitride has a huge thermal conductivity of up to 2000 W/mK, resistance to high-temperature oxidation, and its permittivity value is the lowest among ceramic fillers [106]. In Figure 12d, BN provided the highest increase in *k* up to 1300% from 0.22 to 3.22 W/mK, achieved by highly oriented BN nanoplates [87] (black triangles in Figure 12d). For composites with a homogeneous distribution of BN nanoplates (black squares in Figure 13), the gain in *k* is several times lower, only 60%, from 0.22 to 0.35 W/mK. However, it is still a better result compared to other nanofillers, being at the level of the best Al_2_O_3_ samples. However, if we consider spherical BN nanoparticles [81], then the increase in thermal conductivity will be even lower, up to 55%, from 0.27 to 0.42 W/mK.

In [107], the effect on *k* was investigated for a mixture of fillers, BN, and MWCNTs. A synergistic improvement in thermal conductivity values for them was observed due to the creation of three-dimensional heat transfer paths between BN and MWCNT, and the maximum *k* value was increased from 0.22 to 1.74 W/mK. However, the presence of MWCNTs can cause a drop in electrical resistivity, with the material losing its dielectric properties. The using of BN additives in 3D structures such as polymer aerogels allows it to reach high thermal conductivity [108,109]. In [110], the authors showed excellent in-plane and out-of-plane thermal conductivities of 0.76 W/mK and 0.61 W/mK with a ratio of BN/PVA of (2:1) in comparison with 0.15 W/mK for the pure polyvinyl alcohol (PVA) matrix.

The functionalization or sizing of the surface of nanoparticles is of special importance when dielectric properties are considered [111]. The addition of an organic coating around Barium Titanate nanoparticles reduced agglomeration in polyvinylidene fluoride and caused a decrease in the dielectric constant in comparison with the untreated analog [112]. As another example, the functionalization of the alumina surface makes it electrically conductive [113].

To summarize, dielectric nanoadditives significantly increase the thermal conductivity of the FRPC matrix, leading to better heater performance, with the boron nitride providing the best results. However, the technology has restrictions in the process of FRPC manufacturing—one cannot use more than 10% of the filler caring not to degrade other properties. Bypassing this challenge is possible thanks to using hybrid additives from different fillers and creating 3D thermally conductive networks. The future perspective research should probably focus on hybrid nano additives and 3D network self-assembly and synthesis as applied to heaters for FRPC.

## 4. Self-Heating Polymers

In the previous section, we considered the increase in thermal conductivity of the polymer surrounding the heater, and, to avoid effects such as short-circuiting, electrically conductive nanoadditives were not discussed. However, an increase in the electrical conductivity of a polymer is not always undesirable since in this case the polymer itself can serve as a heating element, and composite products based on such nanoparticles present a separate class of self-heating polymer nanocomposites (PNC) for anti/de-icing.

Electrically conductive nano additives are expected to have high thermal conductivity as well, which is consistent with the Wiedemann–Franz law [114]. Therefore, we generally say that PNCs with electrically conductive nanoadditives shall also possess high thermal conductivity.

A distinctive feature of this type of PNC heater is the ability to apply them as a self-heating coating on the surface of the already manufactured and operating FRPC structure. Since the primary function is heating while the mechanical properties of the coating are assigned a secondary role, there is typically no restriction on the mass percentage of nano additives for PNCs with typical values being well above the percolation threshold.

In Table 2, we compare values of the percolation threshold, electrical conductivity, filler content, and thermal conductivity for the most common polymer nanocomposites with electrically conductive nanoparticles.

The dependence of the experimentally observed percolation thresholds, as of the point where the nanocomposite becomes electroconductive, on the filler type and the matrix material is presented in Table 2 (% value of percolation threshold is either wt. or vol. depending on the value position in the column to the left). The distribution of values agrees with the theoretical predictions by the type of particles and their aspect ratios [157]. According to Table 2, in the sense of increasing nanofiller fraction, the percolation threshold is observed first for 1D filler materials, then for 2D additives, and finally for 0D nanoparticles. However, aligned graphene being a 2D material shows an unrealistic percolation threshold value of 0.03 vol%. This effect is achieved due to the creation of a highly ordered graphene 3D structure using additional additives. In the 3D network graphene case, the percolation threshold is reached by extra-oriented graphene plates [143]. Some authors used electroconductive polymers, such as PDMS [126,130,146], as a matrix. In these cases, the highest value of electrical conductivity is shown, but these polymers are expensive, and it is unprofitable to use them for manufacturing large FRPC.

The dependence from the filler form was also observed for electro and thermal conductivity. Table 2 presents many examples with high and low electro and thermal conductivities, and dependence is the same as for percolation threshold: 1D materials in the first place, then 2D, and finally, 0D, corresponding to the theory described in [157]. This cannot be applied to a 3D network, since in these cases an ordered structure with oriented additives with anisotropic properties is often created. The best results were demonstrated by samples with hybrid fillers from several additive materials [153,155]. The synergy of properties achieves the fusion of multiple effects. The interaction of two or more components in PNC looks very relevant and has great perspectives.

Several PNC compositions are already applied for FRPC heating in practice. In [158] authors studied the application of a PNC heater, based on MWCNTs and acrylic resin, coated onto an FRPC fan blade. When an electrical current was applied, the PNC itself heated up to a heat flux density of 3500 W/m^2^, Figure 13a, and de-icing of the surface of the FRPC blade happened in 300 s working at a rotation of 100 rpm, Figure 13b.

**Figure 14:**
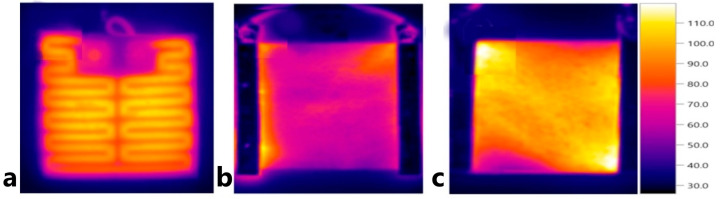
Infrared images of (**a**) NiCr, (**b**) CFs, and (**c**) CFP-2700 heating elements (Reprinted with permission from Elsevier, copyright 2020) [159].

The applicability of short carbon fibers as heaters was demonstrated in [159]. Carbon fibers, chopped at 6 mm, were annealed (graphitized). The change in the structure led to a significant increase in their thermal conductivity and electrical conductivity from 9.8 W/mK and 705.8 S/cm to 153.8 W/mK and 1314.9 S/cm, respectively. Then, PNC heaters were made from the annealed (CF2700) and parent fibers (CFs) with the same fiber content. These heaters were compared to a commercial NiCr heating source, Figure 14. At a voltage of 5 V, the CF2700 heater showed the most uniform heating, the same high temperature (105 °C), and the highest heating rate (400 deg/min).

One study [160] demonstrated the possibility of creating carbon nanotube buckypaper heaters (CNP) based on MWCNTs/epoxy nanocomposite. The samples were manufactured by resin impregnation technology in a vacuum (RV-CNP) and resin impregnation under pressure 0.4 MPa (RP-CNP). When tested for heating, the samples showed the maximal temperature of 110 °C and 130 °C, respectively, which corresponds to a thermal power of 2 kW/m^2^. Tests for de-icing showed high efficiency of RV-CNP, where de-icing took only 120 s.

**Figure 15:**
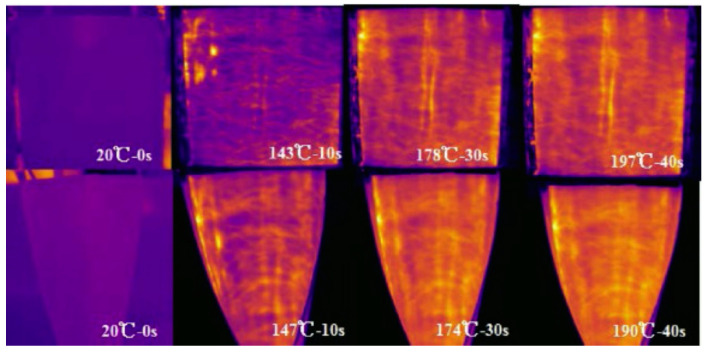
Temperature distribution of G-CF35/PDMS at different times at 35 V voltage not deformed and twisted states (Reprinted from Elsevier, copyright 2019) [161].

Another study showed the possibility of manufacturing flexible self-heating tapes based on commercially available graphene-coated carbon fiber (G-CF35) in a polydimethylsiloxane (PDMS) matrix [161]. The G-CF35/PDMS heater was obtained by spray-coating the part, and tests showed stable results for both not deformed and twisted states with temperatures up to 190 °C and 197 °C, respectively, Figure 15. The maximal reached operating temperature was 297 °C, which corresponded to 11.1 kW/m^2^ of heat flux density.

**Figure 16:**
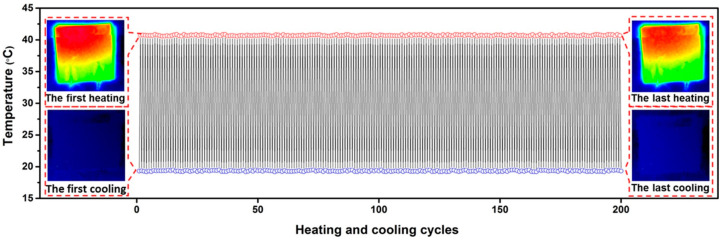
Temperature variation of ceramic heater during thermal cycling using compressed-GF/PDA/APTS/PDMS composite as a thermal interface material (Reprinted with permission from American Chemical Society, copyright 2017) [162].

The synergy of polymer and nanoparticles allows self-heating composites to be produced even in the form of foams. Thus, in [162] a PNC foam was obtained based on a multicomponent composite graphene/polydopamine/3-aminopropyltriethoxysilane/polydimethylsiloxane. The thermal conductivity was found to be highly anisotropic, with 28.77 W/mK in-plane and 1.62 W/mK out-of-plane at 11.62 wt.% graphene loading. The absence of heater degradation was also demonstrated during the cyclic operation of the composite at 40 °C for 200 heating-cooling cycles, Figure 16.

Summarizing, a number of combinations of polymers and electrically conductive nanoadditives have been considered. However, this is only the “tip of the iceberg” when we are considering nanoscale technologies, leaving tremendous potential for future research. The limitation factor here is again the mentioned above the limit of 10% maximum filler content in the matrix.

As we saw before, the optimal performance is provided on one side by coatings, and on another side by PNC solutions. Combining these two technologies into one, polymer nanocomposite coatings allow for the combined benefits of both. For PNC coating, there are no restrictions on filler mass content in comparison to additives in FRPC matrix, and due to the wide range of used materials, almost any functionality can be achieved. So, simultaneously with the heating function, such coatings can provide shielding, be coating condition sensors, have hydrophobic properties, self-healing, etc. This multifunctionality is impossible for metal coatings, even if they are used in conjunction with supporting solutions, presented in the next chapter. We assume that PNC coatings can replace metal coatings in the future. Currently, the factor limiting PNC mass production applications is the high price of most fillers and polymers, but with the development of manufacturing technologies, this problem can be solved in the future.

## 5. Passive Anti/De-Icing Support Solutions

The solutions presented in this chapter can be used both individually and in conjunction with most types of anti/de-icing systems. They were developed long before active systems but are still widely used today. Some of them allow for obtaining good results but unfortunately quickly lose their performance in time. Others have low efficiency but are durable and inexpensive.

### 5.1. Application of Hydrophobic Coatings

Hydrophobic liquids are widely used as materials for passive thin-film coatings for anti/de-icing on the FRPC surface [24,163,164]. Although hydrophobic coatings are convenient to apply and typically not expensive, it is generally not recommended to rely only on a hydrophobic coating alone for anti/de-icing [165]. Moreover, the hydrophobic coating can be destroyed by falling ice, snow, rain, etc. [166].

**Figure 17:**
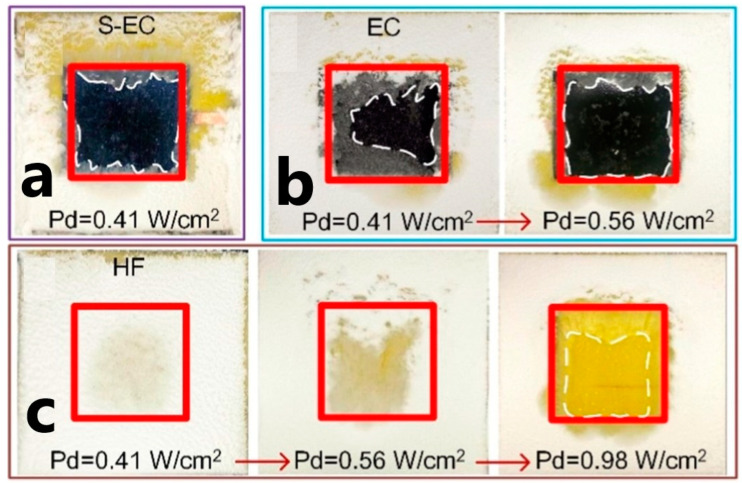
Anti-icing properties of coatings. (**a**) S-EC, (**b**) EC, and (**c**) HF. Areas indicated by red rectangles are heating areas, and white dotted lines are areas without ice (Reprinted with permission from Elsevier, copyright 2018) [158].

In [158,167] authors presented a combined multilayer anti/de-icing effect by applying a superhydrophobic thin-film coating over a PNC heater. Figure 17 compares the results of a de-icing test under identical environmental conditions for three surfaces: the PNC heater combined with a superhydrophobic coating (S-EC), the PNC heater without the superhydrophobic layer (EC), and a polyimide heating film (HF) as a traditional heater. As a result, the S-EC coating coped with the task at a lower energy cost than the EC and HF heaters: 0.41, 0.56, and 0.98 W/cm^2^, respectively.

Moreover, hydrophobic properties can be obtained from the surface, which mimics nature, where hydrophobic properties are often encountered, e.g., lotus leaves, rice, roses, etc. [168,169]. However, these coatings are also not perfect: they are very difficult to obtain in large areas, they have a high cost, and their hydrophobic properties disappear under harsh operating conditions [170].

### 5.2. Chemical Removal

Chemicals preventing water from freezing are used as a treatment in aircraft services. However, when using chemical substances, it is necessary to take into account the pollution of the environment [171] as well as the fact of corrosion. Thereby, for example, this method is not currently used in the operation of turbine blades of wind turbines [172].

### 5.3. Absorbing Sun Radiation

The application of black paint absorbs solar radiation and, thereby, increases the surface temperature, which negatively affects the formation of ice. Black paint is typically used in conjunction with a hydrophobic coating. Black paint heat absorption is not easy to control and can quickly overheat the surface resulting in premature decommissioning of FRPC [173].

## 6. Conclusions

Since the middle of the 20th century, a number of studies have been carried out, aimed at studying the processes of preventing and removing an ice crust from the surface of FRPC products. This area began to develop especially actively with the discovery of new nanosized additives and highly effective hydrophobic compounds. Our study provides an overview of a large number of heating solutions for anti/de-icing on the surface of FRPC products. They can be divided into two groups: external heating systems—located outside the composite product, and internal heating systems—located inside the composite product. However, this division is only formal; for example, the heater based on PNC, an internal heater, can be applied on an FRPC surface and this solution will be named an external heating system. For PNC systems, the percolation threshold is very important. The value of the percolation threshold depends on the aspect ratio of the filler particle [149], the particle’s size [144], the structure of the particles [157], the functionalization of the particle surface [154], and also on their ordering [119]. The FRPC and PNC heaters work more efficiently when accompanied by hydrophobic coatings. The synergistic effect always exceeds the impact of heating and hydrophobic separately [174,175,176].

In general, the effect of the interaction of two or more components in the process of obtaining effective solutions is very relevant. Based on the review, we can conclude that the solutions with the best technical indicators were obtained using multicomponent systems. Thus, the high thermal conductivity of an electrically conductive nanoparticle-polymer composite was obtained with the synergy of graphene and SiC nanowires, 2.13 W/mK at 9.5 wt.% [153]. As for the dielectric case, high-performance *k* was demonstrated by the cellulose nanofiber/NB/epoxy composite, 3 W/mK at 10 wt.%, whereas the thermal conductivity of graphene/epoxy, cellulose nanofiber/epoxy, and NB/epoxy is below 1 W/mK at 10 wt% [87,142]. Similar effects are shown for electrical conductivity and reaching the percolation threshold. In most cases, these phenomena are anisotropic.

To select a technical solution for anti/de-icing the surface of an FRPC product, there is no standard answer; each case requires an individual approach. It is necessary to take into account the characteristics of the used polymer, environment, and reinforcement. When using nano-sized additives, we need to consider their aspect ratio, size, structure, surface functionalization technologies, and peculiarities of interaction with other additives.

## Figures and Tables

**Table 1:** Properties of FRPC heaters.

Type	Tech	Position	Material	Power Density, kW/m^2^	De-Icing time	Anti -Icing	Test Condition	Comments	Ref.
Metal film	Flame-spraying	Surface	NiCr	11.8	-	yes	T = −18 °C	Used on Boeing 787	[32]
Metal Wire	Hand layout	Embedded depth 0.7 mm	UD NiCr wires	8.3	-	yes	T = −17 °C, wind speed 27.7 m/s, water flow 0.2 kg/min	Max applies power 118 W	[40]
	3D printing	Embedded depth 0.6 mm	NiCr wires square meander	10	5 min	yes	T = −20 °C Field study at sea	Max applies power 78 W	[45]
Metal tape	Hand layout	Embedded depth 1 mm	Cu ribbon inside GLARE	26	-	-	At room	Shows interlayers creep effect	[42]
Carbon textile	Hand layout	Embedded depth 0.15 mm	UD brominated graphite fibers	46.5	-	yes	At room	textile resistance 50 μΩ/cm^2^	[39]
	Hand layout	Embedded	Non-woven pure CFMat	6.5	-	yes	At room	Max T = 134 °C after 38 min	[51]
	Hand layout	Embedded	Non-woven CFMat with NiCuNi coating	3.2	-	yes	At room	Max T = 79 °C after 37 min	[51]
	Hand layout	Embedded	CFs with NiP coating	16.8	-	yes	At room	40 s to 100 C	[52]
	Hand layout	Embedded depth 0.15 mm	CF prepreg	6.5	25 s	yes	T = −12 °C	Study at 16 V	[62]
	Hand layout	Embedded depth 2 mm	ECT by “Gorix”	0.854/ 0.929	20 min	yes	T = −20°C + water fog	9.1 V anti/9.9 V de-icing	[55]
	Spray coating	Surface	Non-woven SWCNT mat	7.6	-	yes	At room	Max T = 160 °C after 18 h	[65]
CNT	Roll-to-roll printing	Surface	Non-woven MWCNT mat	1.33	25 min	yes	T = −20 °C and wind speed 7 m/s	Study at 70 V	[57]
	Buckypaper by filtration	Embedded depth 0.5 mm	Non-woven from SWCNT	11	4 min	yes	T = −22 °C and wind speed 0–14 m/s	Study at 22 V	[60]
	Buckypaper by pulling	Embedded depth 0.15 mm	UD from CNT forest	4.9	15 s	yes	T = −12 °C	Study at 16 V	[62]
Graphene	Buckypaper by filtration	Embedded depth 1.115 mm	Non-woven from exfoliated graphite	1.6	4 min	yes	T = −32 °C	Study at 0.8 A	[63]
	Buckypaper by filtration	Embedded	Non-woven from graphene	3.6	210 s	yes	T = −20 °C	Tensile and elastic modulus are growing	[64]
	Spray coating	Surface	Made using graphene nanoribbon	3.88	3 min	yes	T = −20 °C	Study at 177 V	[66]
	Spin coating	Surface	Non-woven from GO	20	30 s	yes	T = −10 °C	Study at 60 V	[67]

**Table 2:** Properties of polymers with electrically conductive nanofillers.

Filler Type	Matrix	ρ at Max %, S/cm	Filler Content, Max %		Experimental Percolation Threshold %	*k* at Max VF, W/mK	Ref.
			Wt.	Vol.			
Carbon black powder	EMA	10^−3^	50	-	18.1	-	[115]
	Epoxol 2004	10^−9^	10	-	5.26	-	[116]
	ED-20 Epoxy	10^−7^	29	-	8	-	[117]
Carbon black nanopowder	PLLA + PDLA	0.1	5	-	2.7	-	[118]
	TPU	0.5	-	10	10.2 wt./6.93 vol	-	[119]
	TPU + COPA	0.3	-	20	5.5 wt./3.68 vol	-	[119]
	Latex + PP + SDBS	0.007	10	-	4.5	-	[120]
	Isotactic PP	0.003	15	-	2	-	[121]
Carbon fiber	CMC	0.014	10	-	4	-	[122]
	YDF-170 Epoxy	0.02	5	-	-	-	[123]
Carbon Nanofiber	Epoxy LY 1564	1.9 × 0^−5^	5	-	-	-	[124]
	Epoxy LY 1564 + BMIMBF4	1.74 × 10^−5^	3	-	-	-	[124]
	DGEBA	5.5 × 10^−4^	1.5	-	0.33	-	[125]
	DGEBA + Gelatin	2 × 10^−5^	1.5	-	0.21	-	[125]
	PDMS	2.1	40	-	-	-	[126]
	CPE	10^−6^	10	-	3	-	[127]
	Polycarbonate	10^−5^	10	-	0.5	-	[128]
MWCNT	Epoxol 2004	10^−4^	10	-	4.71	-	[116]
	Latex + PP + SDBS	0.015	1	-	0.3	-	[120]
	IROGRAN PS 455-203	2. 98 × 10^−2^	40	-	0.2	-	[129]
	PDMS	1	-	4	0.045	0.5 at 1.4%	[130]
	EPOLAM 2031	10^−4^	2	-	-	-	[131]
	TPU	0.1	1	-	0.2	-	[132]
	EPON 828	-	25	-	0.06	-	[133]
	IN2 Epoxy	5.2 × 10^−4^	2.5	-	0.25	-	[134]
	Isotactic PP	8 × 10^−3^	15	-	-	-	[121]
	EPOLAM 2031	5 × 10^−4^	2	-	-	0.22	[135]
	Polypropylene	0.1	-	3	0.8	-	[136]
SWCNTs	EPOLAM 2031	0.5	2	-	-	-	[131]
	Araldite LY 1564 SP	10^3^	10	-	0.08	0.8	[137]
	EPOLAM 2031	0.8	2	-	-	0.5	[135]
Graphite	Latex + PP + SDBS	1.2 × 10^−3^	10	-	7	-	[120]
	EPON 828	-	25	-	12	-	[133]
	PEO	2 × 10^−6^	2	-	-	-	[138]
Graphite nanoplates	Epoxol 2004	10^−13^	10	-	-	-	[116]
Graphene oxide	PVA + PDMS	0.4	1	-	0.42	-	[139]
	PEO	3 × 10^−6^	2	-	-	-	[138]
N_2_ doped graphene	EPOLAM 2031	6 × 10^−12^	2	-	-	-	[131]
Reduced graphene oxide	Latex + PP + SDBS	9.2 × 10^−3^	3.5	-	1.2	-	[120]
	EPOLAM 2031	8 × 10^−12^	2	-	-	-	[131]
	PEO	7 × 10^−5^	2	-	-	-	[138]
Graphene nanosheets	EPON 828	10^−6^	25	-	3.37	-	[133]
	Isotactic PP	2 × 10^−5^	15	-	7	-	[121]
	Araldite F CI	4.63 × 10^−5^	-	2.8	0.8	-	[140]
	Polyethylene	1.3 × 10^−4^	20.9	8.9	8.4 wt/3.8 vol	-	[141]
	E51 epoxy	6 × 10^−6^	-	2	0.63	2.17 at 6%	[142]
Graphene nanosheets	Polyimide (3Dnetwork)	0.94	-	5	0.03	-	[143]
Ag particles	EPON 8281	0.012	-	8	5	0.5082 at 8%	[144]
Ag flakes	Polyurethane	2.9 × 10^−5^	80	30	9	-	[145]
Ag dendrites	Polyurethane	1.8 × 10^−4^	70	20	3	-	[145]
Ag nanoparticles	EPON 8281	0.3	-	8	6	0.366 at 8%	[144]
	PDMS	0.6	-	24	10	1.61 at 24%	[146]
	PVDF	22.9	-	20	6	-	[147]
Ag nanowires	EP/PEI	-	3	-	3	0.3	[148]
	Polystyrene	0.1	30	-	2.4	-	[149]
	DGEBA	4.1	-	8	0.7	1	[150]
Cu nanoparticles	PDMS	0.3	-	24	10	1.34 at 24%	[146]
Cu micro sheets	E51 Epoxy	6 × 10^−3^	80	-	20	-	[151]
Cu nanowires	Polystyrene (3Dnetwork)	80	-	2.8	0.24	-	[152]
	Polypropylene	5	-	3	1.7	-	[136]
Graphene and SiC nanowires	PVDF (3Dnetwork)	0.02	9.5	-	1.5	2.13 at 9.5%	[153]
Ag & Cu particles	PDMS	1.8	-	24	10	1.68 at 14%	[146]
Ag @ Cu flakes	DGEBF	600	70	-	40	-	[154]
Cu nanowires @GO, 3D network	Epon 862	1.2	6	-	-	0.5 at 6% Cu and 1.2% Go	[155]
Graphite + SP + Al_2_O_3_	TPU + PES	0.1	20	-	14.8	-	[156]

## Data Availability

Not applicable.

## Abbreviations

0D	Zero dimensional
1D	One dimensional
2D	Two dimensional
3D	Three dimensional
Al2O3	Alumina
AlN	Aluminum nitride
BMIMBF4	1-n-Butyl-3-methylimidazolium tetrafluoroborate
BN	Boron nitride
CMC	Carboxymethyl cellulose
CNT	Carbon nanotubes
COPA	Copolyamide
CPE	Chlorinated polyethylene
DGEBA	Bisphenol A
EMA	Ethylene methyl acrylate
ECT	Electro-conductive carbon-based textile
FML	Fiber metal laminate
FRPC	Fiber-reinforced polymer composites
GLARE	Glass laminate aluminum reinforced epoxy composite structure
MCF	Metalized carbon fiber
MWCNT	Multi-wall carbon nanotubes
NiCr	Nickel-chromium
PEO	Polyethylene glycol
PES	Polyethersulfone
PDLA	Polylactide
PDMS	Polydimethylsiloxane
PLLA	Poly(L-lactide)
PNC	Polymer nanocomposites
PP	Polypropylene
PVA	Polyvinyl alcohol
PVDF	Polyvinylidene fluoride
SBR	Styrene-butadiene rubber
SiC	Silicon carbide
SDBS	Sodium dodecyl benzene sulfonate
TPU	Thermoplastic polyurethane
